# In-Depth Analysis of Patient-Clinician Cell Phone Communication during the WelTel Kenya1 Antiretroviral Adherence Trial

**DOI:** 10.1371/journal.pone.0046033

**Published:** 2012-09-25

**Authors:** Mia L. van der Kop, Sarah Karanja, Lehana Thabane, Carlo Marra, Michael H. Chung, Lawrence Gelmon, Joshua Kimani, Richard T. Lester

**Affiliations:** 1 University of British Columbia, Vancouver, British Columbia, Canada; 2 British Columbia Centre for Disease Control, Vancouver, British Columbia, Canada; 3 University of Manitoba, Nairobi, Kenya; 4 McMaster University, Hamilton, Ontario, Canada; 5 University of Washington, Seattle, Washington, United States of America; 6 University of Nairobi, Nairobi, Kenya; University of Pennsylvania, United States of America

## Abstract

**Background:**

The WelTel Kenya1 trial demonstrated that text message support improved adherence to antiretroviral therapy (ART) and suppression of HIV-1 RNA load. The intervention involved sending weekly messages to patients inquiring how they were doing; participants were required to respond either that they were well or that there was a problem.

**Objectives:**

1) Describe problems participants identified through mobile phone support and reasons why participants did not respond to the messages; 2) investigate factors associated with indicating a problem and not responding; and 3) examine participant perceptions of the intervention.

**Design:**

Secondary analysis of WelTel Kenya1 trial data.

**Methods:**

Reasons participants indicated a problem or did not respond were extracted from the study log. Negative binomial regression was used to determine participant characteristics associated with indicating a problem and non-response. Data from follow-up questionnaires were used to describe participant perceptions of the intervention.

**Results:**

Between 2007 and 2009, 271 participants generated 11,873 responses; 377 of which indicated a problem. Health issues were the primary reason for problem responses (72%). Rural residence (adjusted incidence rate ratio [IRR] 1.96; 95%CI 1.19–3.25; p = 0.009 and age were associated with indicating a problem (adjusted IRR 0.63 per increase in age group category; 95%CI 0.50–0.80; p<0.001). Higher educational level was associated with a decreased rate of non-response (adjusted IRR 0.81; 95%CI 0.69–0.94; p = 0.005). Of participants interviewed, 62% (n = 129) stated there were no barriers to the intervention; cell phone issues were the most common barrier. Benefits included reminding patients to take medication and promoting a feeling that “someone cares”.

**Conclusions:**

The WelTel intervention enabled frequent communication between clinicians and patients during the WelTel Kenya1 trial. Many patients valued the service for the support it provided, with health-related concerns comprising the majority of problems identified by participants. Few sociodemographic characteristics were associated with participant engagement in the intervention.

## Introduction

The NIH Consensus Group defines mobile health (mHealth) as the use of mobile and wireless devices to improve health outcomes, healthcare services and health research. [1] The global expansion in cell phone use, with high rates of uptake in Africa, presents new opportunities to incorporate mHealth into health service delivery in resource-poor settings. Our recent randomized controlled trial (WelTel Kenya1) demonstrated that cell phone text messages significantly improved adherence to antiretroviral therapy (ART) and suppression of HIV-1 RNA load. [2] The trial’s intervention, a weekly text message “Mambo?” (Kiswahili for “How are you?”), required active participation on behalf of intervention arm participants in the form of a response either that they were well or had a problem. While the original results of the trial have been published, [2] they did not include a detailed examination of patient-clinician communication arising through the intervention or a thorough account of patient perceptions of the service.

Research of mHealth applications in HIV/AIDS care is limited but growing, with a key area being support for those on treatment. Of the studies conducted thus far, cell phones have been used as a medication reminder (timed with dosing schedules), [3], [4], [5], [6] an adherence-reporting tool, [5], [7], [8] and as a mechanism to support patients. [9] Although some text-messaging adherence interventions for HIV/AIDS and other patients have not required participants to respond, [3], [4] most have required some type of patient response. [5], [6], [7], [10], [11] Research of patient characteristics associated with participant engagement in text-message interventions is limited. [12], [13] The objectives of this study were to: 1) describe problems participants identified through patient-clinician cell phone communication and reasons why participants did not respond to the texts; 2) investigate factors associated with non-response and indicating a problem; and 3) describe participants’ perceptions of the barriers to and the benefits of the WelTel service.

## Methods

### Study Design and Population

WelTel Kenya1 was an individually randomised, multi-site controlled trial. Patients initiating ART were recruited from three HIV clinics involved in ART provision scale-up: the Pumwani Clinic serving a low-income population in Nairobi; the Coptic Hope Center for Infectious Diseases operating out of a faith-based hospital in a higher-income area of Nairobi; and the Kajiado Clinic, a government health centre in a large rural district. Patients were eligible for study participation if they were over 18 years old, initiating ART, had access to a mobile phone, and could communicate through text-messaging.

Between May 2007 and October 2008, 538 patients were randomised: 273 to the intervention arm and 265 to standard care. Only participants in the intervention arm are included in this report. Full details of the WelTel Kenya1 trial design and population have been published previously. [2], [14] Written informed consent was obtained from all participants. The original study protocol was approved by the University of Manitoba and Kenyatta National Hospital ethics review boards.

### Intervention

For 12 months, every Monday morning, a clinician (nurse) sent the text message “Mambo?” (“How are you?”) to patients in the intervention group to inquire about their status. Patients were instructed to respond within two days either that they were doing well (“Sawa.”) or had a problem (“Shida.”). The clinician called and provided triage to patients who indicated a problem or failed to respond. Non-response was defined as not responding within 48 hours of the message. All responses, instances of non-response, and mobile phone communications between the health care providers and patients were manually recorded in a study log. Data was entered in Microsoft Access on a weekly basis.

### Study Outcomes

#### Problem responses

Reasons for problem responses were categorized as health or non-health issues. Health issues were further grouped according to the system affected (e.g. gastrointestinal, respiratory, etc.); or as general malaise; oedema; loss of appetite; in hospital (patient reported they were admitted to hospital); or other (e.g. dizziness, depression). Unspecified health issues included reports of a medical nature that were too general to be further classified. Non-health reasons for a problem response included logistical issues or personal problems. Logistical issues were related to: i) cell phone use; ii) appointments; or iii) medication (e.g. lost or stolen medication).

#### Non-response

Reasons why participants did not respond to the text messages were broadly grouped as cell phone problems or factors relating to the participant. Participant factors included forgetting to text back, being too busy, travelling, health or personal reasons, not understanding the protocol, the patient was recently seen (or was going to be seen) at the clinic, or other (e.g. participant tried to call the clinic). The reason for non-response was unavailable in instances where the participant responded late (48 hours after the message was sent but before the clinician called the patient back), the participant was unreachable, the reason was not specified (although contact was made), the participant reported having replied, or data was missing.

#### Factors associated with response type

There have been few reports on the association between participant characteristics and response to text-messaging interventions, minimizing our ability to base variable selection on *a priori* data. Studies reporting this type of information have generally found that characteristics are *not* associated with participant response; [12], [13] however, differences in the study populations, settings, health condition targeted, and interventions led us evaluate factors previously found not associated with participant response. We included basic demographic characteristics: sex; age; marital status (single, married, or separated/divorced/widowed); residence (rural or urban); and highest educational level attained (no formal education; primary; secondary; post-secondary). In addition, we hypothesised that duration of participation in the intervention; clinic attended; disclosure of HIV status (to 0 persons; 1 person; 2–4 persons and ≥5 persons) and whether a participant used their own phone or somebody else’s to respond to the messages may be associated with participant response. Instances of non-response due to messages sent in error (after participant death or withdrawal) were excluded from this analysis.

#### Participant-perceived barriers and benefits

Interviewer-administered questionnaires within three months of the final 12-month study visit were used to ascertain participant perceptions of the intervention. Answers to open-ended questions on what participants believed was the biggest barrier to and greatest benefit of the WelTel service were categorized and described.

#### Statistical analyses

Descriptive analyses of the study population, participant perceptions, and reasons for problem- and non-response were conducted in SPSS v14. Frequencies of the reasons underlying each response type were tabulated as the number of total responses and the number of unique responses (which excluded responses by the same participant for the same reason in subsequent weeks). The proportion of problem responses and non-responses per quarter-year was calculated using a denominator of all messages sent during the same period. A chi-squared test was used to determine whether the proportion of problem- and non-response differed between time periods.

Our count data were overdispersed relative to the Poisson distribution; therefore, we analysed the data using negative binomial regression models. Estimated incidence rate ratios are presented with corresponding 95% confidence intervals (CI) and p-values for the explanatory variables against the outcome of i) a problem response and ii) non-response. An exposure variable was incorporated in all models to account for variability in how long participants participated in the intervention. Univariable analyses were performed first to assess the strength of the association between each factor and the outcome. Factors were then included in the multivariable model if we had hypothesized that they would be of significance (i.e. duration of participation) or if they had a significant univariable p-value (≤0.10). In the final models, variables (other than duration of participation) were selected based on a significance threshold of p<0.05. All p-values are two-sided and reported to three decimal places with those less than 0.001 reported as p<0.001. To determine whether to include a linear effect or indicator variables for ordered categorical variables, nested models were compared using the likelihood ratio test. Interaction between variables was examined by stratification and tested with the likelihood ratio test. A manual backwards stepwise procedure was used to confirm the models. Analyses were performed using Stata version 12 (Statacorp, College Station, TX).

## Results

### Study Population

Of 273 intervention arm participants, two did not receive any messages (one died and the other lost phone access before receiving the intervention). Characteristics of the remaining 271 participants are shown in Table 1. The majority of participants were married, female, living in an urban area, used their own phone to send and receive messages, and at baseline, had disclosed their HIV status to one person.

**Table 1:** Characteristics of the 271 patients who participated in the SMS intervention during the WelTel Kenya1 trial.

Characteristic	Number (%)
**Sex**	
Female	176 (65)
Male	95 (35)
**Age group (years)**	
19–29	56 (21)
30–39	132 (49)
40–49	60 (22)
≥50	23 (8)
**Highest educational level attained**	
None	9 (3)
Primary	108 (40)
Secondary	106 (39)
Post-secondary	48 (18)
**Marital status**	
Married	142 (52)
Single	52 (20)
Separated/divorced/widowed	77 (28)
**Residence**	
Rural	50 (18)
Urban	221 (82)
**Clinic**	
Pumwani	119 (44)
Coptic	117 (43)
Kajiado	35 (13)
**Phone used to send and receive messages**	
Own phone	220 (81)
Somebody else’s phone	51 (19)
**Disclosure of HIV status (number of people)**	
0	37 (14)
1	118 (44)
2–4	83 (30)
≥5	33 (12)

### Text Messages Sent and Received

Between June 2007 and November 2009, 11,885 “Mambo?” messages were sent to participants. Of 11,873 (99.9%) documented responses, 377 (3%) were ‘shida’ (problem); 7,766 (65%) were ‘sawa’ (OK); and 3,730 (31%) were instances of non-response. The numbers of problem responses and instances of non-response categorized by participant characteristic are shown in Table 2. Participants received the messages for a median duration of 50 weeks (interquartile range, 40–52 weeks). In the analysis of factors associated with response type, 106 non-responses due to messages sent after death (n = 49) or withdrawal (n = 57) were excluded. The non-response rate modestly increased from 30% (1027/3,429) in the first 3-months of participation to 31% (1017/3,274); 33% (966/2,958) and 33% (45/2,212) in subsequent quarter-years respectively (p = 0.071). Conversely, the proportion of problem responses decreased from 5.9% (203/3,429) to 2.4% (79/3,274); 1.7% (50/2,958) and 2.0% (45/2,212) in subsequent quarter-years respectively (p<0.001).

**Table 2:** The number of problem responses and instances of non-response during the WelTel Kenya1 trial.

	Problem response					Non-response				
Characteristic	No.	Mean	SD*	Median	IQR	No.	Mean	SD*	Median	IQR
**Sex**										
Female	252	1.43	2.55	1	0–2	2592	15.07	11.83	13	5.5–22.5
Male	125	1.32	2.11	1	0–1	1032	11.35	12.40	7	2–16
**Age group (years)**										
19–29	129	2.30	3.91	1	0–2.5	877	15.95	12.39	13	5–27.5
30–39	170	1.29	1.84	1	0–2	1813	13.97	12.39	10.5	4–19
40–49	70	1.17	1.74	1	0–2	671	11.77	9.86	10	3.5–18
≥50	8	0.35	0.78	0	0–0	263	12.48	14.98	8	2–14
**Level of education**										
None	14	1.56	2.24	1	0–1	125	14.00	12.63	10	3–19
Primary	163	1.51	3.03	1	0–2	1619	15.38	12.46	13	5.5–20.5
Secondary	127	1.20	1.81	0	0–2	1455	14.29	12.50	11.5	4–23
Post-secondary	73	1.52	1.95	1	0–2	425	8.92	9.27	6.5	2–13
**Marital status**										
Married	187	1.32	2.05	1	0–2	1807	13.20	12.10	10	3–19
Single	84	1.62	2.35	1	0–2	590	11.79	10.63	8	4–17
Sep./div./wid.	106	1.38	2.98	0	0–2	1227	16.14	12.92	13	5–26
**Residence**										
Rural	80	1.60	2.17	1	0–2	623	12.50	10.99	11	4–18
Urban	297	1.34	2.45	0	0–2	3001	14.05	12.39	11	4–21
**Clinic**										
Pumwani	184	1.55	2.92	1	0–2	2011	17.40	13.23	14	7–27
Coptic	138	1.18	1.73	0	0–2	1120	9.95	9.70	7	2–13
Kajiado	55	1.57	2.39	1	0–3	493	14.14	12.06	11	4–20
**Phone access**										
Own phone	277	1.26	1.87	1	0–2	2784	13.10	12.09	9.5	3–19
Shared phone	100	1.96	3.93	1	0–2	840	16.63	12.06	13	8–24
**Disclosure**										
0	44	1.19	1.52	1	0–2	539	15.59	14.62	10	4–29
1	160	1.36	2.13	1	0–2	1714	14.87	12.62	13	3–23
2–4	129	1.55	3.18	1	0–2	940	11.61	10.61	9	4–18
≥5	44	1.33	1.81	1	0–2	431	13.15	10.56	12	7–19

* standard deviation.

### Reasons for Indicating a Problem

Approximately half (52%) of the participants who received the intervention responded with a problem at least once during the trial (Table 3). Health issues (72%) were the predominant reason for indicating a problem (Table 3). Gastrointestinal illness and general malaise were the most common health complaints. Non-health issues, most of which where logistical in nature, resulted in 11% of problem responses. Personal problems, cited 3% of the time, included reports of domestic abuse, job loss, and concerns regarding sick family members. In 18% of problem responses, the reason for indicating a problem was unavailable, primarily because the participant was unreachable (9%) when the clinician tried to contact them, or because of missing data (7%). In all of these instances, participants (or a relative) were in subsequent contact with the clinic.

**Table 3:** Reasons for responding with a problem to the weekly SMS.

Reason	Problem responses* §	Unique problem responses* ^∧^
Total	377 (100)	140 (100)
**Health issues**	**272 (72)**	**121 (86)**
Gastrointestinal (abdominal pain, vomiting, etc.)	67 (18)	49 (35)
General malaise	68 (18)	40 (30)
Neurological (headache, back pain, etc.)	60 (16)	42 (28)
Respiratory (coughing, chest pain, dyspnea, etc.)	57 (15)	41 (29)
Dermatological (rash, itching, blisters, etc.)	43 (11)	21 (15)
In hospital	21 (6)	13 (9)
Oedema	12 (3)	9 (6)
Loss of appetite	11 (3)	9 (6)
Genitourinary (genital sores, discharge, etc.)	6 (2)	6 (4)
Other (palpitations, vision problems, etc.)	31 (8)	28 (20)
Other – unspecified	7 (2)	7 (5)
**Non-health issues**	**40 (11)**	**33 (24)**
Personal	13 (3)	11 (8)
Logistical – medication-related	13 (3)	11 (8)
Logistical – cell phone-related	9 (2)	9 (6)
Logistical – appointment-related	7 (2)	7 (5)
**Data unavailable**	**67 (18)**	**50 (36)**
Unreachable	32 (9)	22 (16)
Missing	27(7)	23 (16)
Unable to discuss	8 (2)	5 (4)

* Figures are numbers (percentages). Percentages do not sum to 100 because of non-mutually exclusive response categories.

§ Includes repeat problems (i.e. includes problems indicated by the same participant on more than one occasion in response to the outgoing weekly “Mambo?” text message).

**∧**Excludes repeat problems (i.e. only includes the first time a participant reported a particular problem; excludes reports of problems by the same participant for the same reason in subsequent weeks).

### Factors Associated with a Problem Response

In both univariable and multivariable analyses, problem responses were linearly associated with age, with the adjusted rate of a problem response decreasing by a factor of 0.63 (95% CI 0.50–0.80; p<0.001) per increase in age group category (Table 4). Attending the rural Kajiado Clinic and shared phone access were positively associated with indicating a problem in the univariate analyses; however, after mutual adjustment, these factors were no longer significant. The rate of problem response was greater among participants living in a rural area (adjusted IRR 1.96; 95% CI 1.19–3.25; p = 0.009).

**Table 4:** Associations between covariates and problem responses to text messages during the WelTel Kenya1 randomized controlled trial.

	Unadjusted (univariable) incidence rate ratios (IRR)			Adjusted* (final model) incidence rate ratios (IRR)		
Factor	IRR	95% CI	P value	IRR	95% CI	P value
**Female sex**	0.98	(0.65–1.48)	0.929			
**Age group** †	0.66	(0.52–0.83)	<0.001	0.63	(0.50–0.80)	<0.001
**Education** †	0.90	(0.71–1.14)	0.394			
**Marital status**						
Married	1.00	Referent				
Single	1.20	(0.72–2.01)	0.486			
Sep/div/wid	0.94	(0.60–1.46)	0.771			
**Rural residence**	1.77	(1.06–2.95)	0.030	1.96	(1.19–3.25)	0.009
**Clinic**						
Coptic	1.00	Referent				
Pumwani	1.37	(0.88–1.95)	0.133			
Kajiado	2.51	(1.32–4.76)	0.005			
**Shared phone**	1.90	(1.17–3.07)	0.009			
**Disclosure** †	1.05	(0.84–1.32)	0.675			

* Final regression model mutually adjusted for all significant (p<0.05) covariates. Female sex, education, marital status, and disclosure were not entered into the multivariable model. Clinic and shared phone access did not significantly add to the model and were therefore excluded.

† IRR corresponds to a decrease in the incidence rate ratio per unit increase in education and age category and an increase in the incidence rate ratio per unit increase in disclosure category.

### Reasons for Not Responding

Of 271 participants who received the intervention during the trial, 260 did not reply to a message on at least one occasion. Cell phone problems (22%) were the most commonly cited reason for not responding, mostly due to a lack of network credit (Table 5). Forgetting to text back was cited as a reason for not responding in 6% of the instances of non-response. In most cases, the reason for non-response could not be ascertained (62%), primarily because the participant was unreachable when the clinician tried to contact them. Of these participants, 89% (n = 191) were in subsequent contact with the clinic, either via text message, by phone, or in person.

**Table 5:** Reasons for non-response.

Reason participant did not respond	Non-responses	Unique participant
	n = 3730	non-responses
	n (%)* §	n = 260
		n (%)* ∧
**Cell phone problems**	**820 (22)**	**205 (79)**
Lack of credit	456 (12)	136 (52)
Issues with shared phone access	79 (2)	32 (12)
Phone not functioning properly	65 (2)	40 (15)
Did not receive the message	56 (2)	46 (18)
Owner did not have the phone	48 (1)	36 (14)
Network problems	41 (1)	31 (12)
Battery was not charged	35 (1)	29 (11)
Phone lost/stolen/sold	22 (1)	20 (8)
Difficulties operating the phone/texting	18 (1)	14 (5)
**Participant factors**	**463 (12)**	**172 (66)**
Forgot to respond	207 (6)	108 (42)
Too busy to respond	57 (2)	34 (13)
Travelling	53 (1)	45 (17)
Recently seen (or will be seen) at clinic	43 (1)	37 (14)
Health issues	46 (1)	29 (11)
Personal issues	22 (1)	20 (8)
Did not understand the protocol	14 (<1)	11 (4)
Other	21 (1)	18 (7)
**Messages sent after study exit**	**106 (3)**	**19 (7)**
Withdrawal from study	57 (2)	7 (3)
Death	49 (1)	12 (5)
**Data on reason unavailable**	**2314 (62)**	**232 (89)**
Participant unreachable	1932 (52)	214 (82)
Reports to have replied	83 (2)	61 (24)
Reason not specified	49 (1)	32 (12)
Participant responded late	14 (<1)	14 (5)
Missing data	236 (6)	112 (43)

* percentages do not sum to 100 because of non-mutually exclusive response categories.

§ Includes repeat reasons for non-response (i.e. includes reasons for non-responses indicated by the same participant on more than one occasion when they did not respond to the outgoing weekly “Mambo?” text message).

∧ Excludes repeat reasons for non-response (i.e. only includes the first time a participant reported a particular reason for not responding; excludes non-response on subsequent occasions by the same participant for the same reason).

### Factors Associated with Non-response

In the univariable analysis, clinic attended, shared phone access, and educational level were associated with non-response (Table 6), while females had a borderline significant increased rate of non-response compared to males. (IRR 1.27; 95% CI 1.00–1.61; p = 0.055). When adjusted IRRs were calculated for each level of education, a decreased rate of non-response was significant among those with a post-secondary level of education (adjusted IRR 0.81 per unit increase in category of education; 95% CI 0.65–0.94; p = 0.005) (Table 6). The rate of non-response was also associated with the clinic attended. Non-response rates were similarly elevated among participants at the Pumwani and Kajiado clinics compared to those attending the Coptic clinic (adjusted IRRs 1.64; 95% CI 1.30–2.08; p<0.001 and 1.65; 95% CI 1.15–2.37; p = 0.006 respectively). Shared phone access was not a significant factor in the final multivariable model.

**Table 6:** Associations between covariates and non-response to text messages during the WelTel Kenya1 randomized controlled trial.

	Unadjusted (univariable) incidence rate ratios (IRR)			Adjusted* (final model) incidence rate ratios (IRR)		
Factor	IRR	95% CI	P value	IRR	95% CI	P value
**Female sex**	1.27	(1.00–1.61)	0.055			
**Age group** †	0.93	(0.81–1.06)	0.266			
**Education** †	0.75	(0.65–0.87)	<0.001	0.81	(0.69–0.94)	0.005
**Marital status**						
Married	1.00	referent				
Single	0.95	(0.70–1.30)	0.748			
Sep/div/wid	1.16	(0.89–1.51)	0.260			
**Rural residence**	1.03	(0.76–1.39)	0.836			
**Clinic**						
Coptic	1.00	referent		1.00	referent	
Pumwani	1.75	(1.38–2.21)	<0.001	1.64	(1.30–2.08)	<0.001
Kajiado	1.89	(1.33–2.70)	<0.001	1.65	(1.15–2.37)	0.006
**Shared phone**	1.47	(1.10–1.96)	0.009			
**Disclosure** †	0.92	(0.81–1.05)	0.223			

* Final regression model mutually adjusted for all significant (p<0.05) covariates. Age group, marital status, rural residence, and disclosure were not entered into the multivariable model. Female sex and shared phone did not significantly add to the model and were therefore excluded.

† IRR corresponds to a decrease in the incidence rate ratio per unit increase in education, age and disclosure category.

### Patient-perceived Barriers and Benefits

Follow-up questionnaire data was available for 205 (75%) participants. Of those who responded to the question on the greatest benefit of the service (194/205), the most common response was that it reminded them to take their medication (54%). Participants also reported that it reminded them to keep their appointments (14%) and take care of their health (4%). Feeling that “somebody cares” featured as a benefit (22%), as well as being able to access medical advice and report side-effects or health problems quickly (10%). The following were also cited as the greatest benefit but constituted 5% or less of responses: feeling encouraged, hopeful, decreased feelings of isolation, communication with a healthcare provider, and time and cost savings of not having to come to the clinic.

When asked what the greatest barrier was to the intervention, 129 (66%) respondents stated that there were no barriers to the service. When participants indicated a barrier, cell phone-related barriers were most common, with a lack of network credit most frequently cited (20%). Other cell phone issues included keeping the battery charged (3%), network problems (2%), losing access to the phone (3%), and issues with sharing a phone (2%). Only eight participants (4%) indicated barriers unrelated to cell phone use, including fear of disclosure noted by four participants (2%). Responses to the barrier question were missing in 9 instances, resulting in an overall response rate of 72% (n = 196/271).

## Discussion

### Principal Findings

This follow-up analysis to the WelTel Kenya1 trial found that health-related concerns were the primary reason participants indicated they had a problem. Compared to the first three months of participation, participants were less likely to report a problem in subsequent quarters. The change in non-response over time was less evident. Non-response to the WelTel messages was largely temporary as most participants responded in subsequent weeks. In the majority of instances of non-response, the reason why participants did not respond could not be ascertained; when it was, cell phone problems were frequently cited. The rate of non-response varied between clinics, which may reflect unmeasured differences in provider or patient characteristics, attitudes, or the provision of care through the service. Many participants felt there were minimal barriers to the intervention. Perceived benefits of the service included reminding patients to take their medication and feeling that “someone cares”.

### Strengths and Limitations of the Study

This is the first in-depth investigation of an effective cell phone text-messaging adherence intervention that includes a detailed examination of patient-clinician communication, factors associated with response, and patient perceptions of the intervention. Strengths of this study include its long duration, relatively low rate of loss to follow-up, and large number of participants. Three different clinical sites were involved in the trial; however, this study may still be limited in its generalizability to higher-resource settings or different cultures. Each mHealth intervention has unique features, and findings in this population in these particular settings, under clinical trial conditions, may not be applicable in other populations.

This study utilized highly complete, prospectively collected data on participant characteristics and cell phone communication between clinicians and participants. Data on participants’ reasons for non-response, however, were largely unavailable because the majority of participants were unreachable at the time clinicians called them back. If the reasons for not responding differed between participants who could be contacted and those who could not, our findings may be biased. However, we were able to ascertain the reason for non-response on at least one occasion for over 90% (244/260) of participants who did not respond, ensuring a broad representation of non-responders in the reasons presented. Another limitation of our study is the possible influence of social desirability bias in data collected during interviewer-administered questionnaires on patient perceptions of the service. During the original trial, we attempted to minimize this bias through rigorous and standardized training of study personnel. In this study, we confirmed participants’ answers on open-ended questionnaire data by cross-checking responses with additional, related, closed-ended questions embedded in the questionnaire. The response rate to the follow-up questionnaire was 72%; therefore, potential non-response bias may have influenced our findings on participant perceptions.

### Comparison with Other Studies

Pop-Eleches *et al.’s* large trial in Western Kenya also demonstrated the effectiveness of a weekly text-message ART adherence intervention; [9] however, their intervention consisted of one-way text-messages reminding or supporting patients versus the two-way messaging used in our trial. As a result, there is a lack of data from that trial to which we can compare the results of this study. In our study, response rates diminished marginally over a one year period (30–33%). A much greater risk of non-response over time was found in two smaller, shorter trials that used two-way text messages as ART reminders, despite the use of a reminder mechanism if a participant did not initially respond in those studies. [5], [6], [12] Differences between the interventions may explain the differences in the results: one trial used a pager and both required responses to more frequent messages, which posed additional participant burden compared to once-weekly texts and perhaps resulted in greater participant fatigue. Rather than requiring a personalized, more general response, participant response in the other interventions was to acknowledge receipt of the reminder text or to confirm that the participant had taken their medication, potentially contributing to the variability in the sustainability of response rates.

The rate of problem response was markedly different between the first three months of participation and the remainder of the study. We believe this was because the first three months of the study coincided with the period during which the most disease instability would be expected among patients initiating ART. [15] Overall, the problem response rate per week was low (3%), alleviating concerns by program health providers that work load could substantially increase. The study nurses reported that this allowed them to focus on patients who were most in need of support. A similar rate was found in a small pilot study of the WelTel intervention among participants initiating treatment for latent tuberculosis therapy in British Columbia, Canada, in which the problem response rate was 4% during the first 12-weeks of the intervention (unpublished data).

A two-way pager-based messaging study that investigated associations between participant characteristics and non-response similarly found that few patient characteristics were associated with response rates. [12] Unlike our study, the pager study did not find an association between education and non-response. The decreased rate of non-response among those with a post-secondary education may reflect differences in cell phone use [16], [17] or health service utilization in the Kenyan population. [18] Despite a gender gap in Kenya with respect to using cell phones to SMS, [16], [19] we did not find that sex was significantly associated with either non-response or problem response; however, the direction of effect (increased rate of non-response among females) is consistent with the literature. [15].

The WelTel intervention enabled participants to report problems that they were experiencing related to their medication or otherwise. Most other text-messaging interventions have not been designed to elicit these types of responses; however, one of the first studies of messaging to improve ART adherence included text messages to participants inquiring about side-effects. [11] Similar to this study, the most common side-effects reported related to gastrointestinal illness. The health-related nature of the majority of problems reported points towards the potential usefulness of the service for patients to seek advice without having to surmount barriers to in-clinic follow-up, including distance to clinic and transportation. Although we lack data to draw firm conclusions, this may have been a factor in why rural versus urban residents were more likely to report a problem. Despite evidence that side-effects of ART may be more common among older patients, [20] participants in younger age groups were more likely to report a problem. This may reflect subtle age-related differences in comfort levels reporting problems to a healthcare provider over a cell phone, comfort levels reporting a problem in general, a true increased incidence of problems among younger age groups, or another factor not yet determined.

Despite the disparate nature of mHealth ART adherence interventions so far, the majority of studies report that text-messaging interventions were well-received by patients. Similar to patients in the WelTel trial, many participants found messages “highly useful” and that messaging helped remind them to take their medication. [4], [11] Exceptions to this include a recent study by Sidney *et al*, in which participants found weekly interactive voice response messages preferable to pictorial SMS messages. [7] In contrast, a qualitative study from Peru found that participants preferred the idea of text-messages versus recorded voice messages. [21] Interestingly, despite the fact that the outgoing WelTel message, ‘Mambo?’, was not a motivational message *per se*, many of the positive aspects ascribed to motivational messages that appealed to participants in the Peruvian study were perceived as benefits of WelTel, including a feeling that “somebody cares about me” and decreased feelings of loneliness.

This study found that many participants felt there were no barriers to using the text-messaging intervention. Of barriers cited, cost was a concern, which is consistent with findings from an American study, [5] and may pose an even greater barrier in interventions where frequent responses are required. Unlike our study, a significant barrier in a recent study from South India was respondents not knowing how to use text-messaging. [7] Pop-Eleches *et al.* did not specifically examine patients’ perceptions of barriers; however, they did find that phone number changes, lost phones, and network outages did not impede the success of the weekly intervention in improving adherence. [9] Despite highly competitive price points for cell phone services in resource-limited settings in Africa, cost needs to be considered. Effort is needed to find sustainable scale-up strategies to ensure that individuals who might benefit from such a service will not be excluded.

### Conclusions

Effective ART adherence interventions are critical to maximize the individual and population-level benefits of HIV/AIDS control. This study presented a comprehensive analysis of patient-clinician communication during the WelTel Kenya1 trial of an effective text-messaging adherence intervention. Subsequent controlled trials in expanded settings and in-depth qualitative research are required to confirm and elucidate findings on the factors associated with participant engagement and the durability of participation in WelTel and other similar interventions.
